# *l*-Menthol increases extracellular dopamine and c-Fos-like immunoreactivity in the dorsal striatum, and promotes ambulatory activity in mice

**DOI:** 10.1371/journal.pone.0260713

**Published:** 2021-11-30

**Authors:** Toyoshi Umezu, Tomoharu Sano, Junko Hayashi

**Affiliations:** Health and Environmental Risk Division, National Institute for Environmental Studies, Tsukuba, Ibaraki, Japan; Universidade do Estado do Rio de Janeiro, BRAZIL

## Abstract

Similar to psychostimulants, the peripheral administration of menthol promotes mouse motor activity, and the neurotransmitter dopamine has been suggested to be involved in this effect. The present study aimed to elucidate the effects of *l*-menthol on parts of the central nervous system that are involved in motor effects. The subcutaneous administration of *l*-menthol significantly increased the number of c-Fos-like immunoreactive nuclei in the dorsal striatum of the mice, and motor activity was promoted. It also increased the extracellular dopamine level in the dorsal striatum of the mice. These observations indicated that after subcutaneous administration, *l*-menthol enhances dopamine-mediated neurotransmission, and activates neuronal activity in the dorsal striatum, thereby promoting motor activity in mice.

## Introduction

Menthol, which is also known as mint camphor, is a monocyclic terpene alcohol that is found naturally in more than 100 essential oils, and it is a major constituent of the essential oils of spearmint and peppermint. Due to its minty flavor and cool sensation, menthol is among the world’s most widely used flavoring agents; it is used in a variety of commercial products, such as cosmetics, oral hygiene products, including toothpaste and mouthwash, chewing gum, confectionary, foods, beverages, liqueur, cigarettes, and pharmaceuticals. It is estimated that between 30,000 and 32,000 metric tons of menthol is consumed annually [1, 2].

Previous studies have suggested that menthol has effects not only peripherally, but also on the central nervous system (CNS). An experiment using a tilting-cage method, that is more sensitive to horizontal movement such as locomotion than to vertical movement, revealed that the peripheral administration of *dl*-menthol promotes motor activity, i.e., ambulatory activity, in mice [3]. Given that this behavior-activating effect is similar to that of known psychostimulants but distinct from that of nicotine and CNS depressants [4–6], the ambulatory effect of *dl*-menthol has been suggested to result from the activation of some part of the CNS. However, the CNS-activating effects of menthol have not yet been fully elucidated. Thus, the present study aimed to explore the CNS-activating effects of menthol. There are several isomers of menthol, such as *l*-menthol (or (-)-menthol) and *d*-menthol (or (+)-menthol). Stereoselectivity has been suggested for some of the effects of menthol on known target molecules, and *l*-menthol retains better cooling properties than *d*-menthol [7]. As the main isomer found in nature is *l*-menthol, the present study explored the CNS-activating effects of *l*-menthol.

The subcutaneous administration of *dl*-menthol at 400 to 800 mg/kg had significant ambulation-promoting effects in mice [8]. Although previous *in vitro* studies have shown that menthol can influence neuronal activities in some brain parts [9–11], the *in vivo* effects of menthol on neuronal activities in the brain regions involved in locomotion remain unclear. Thus, the present study primarily aimed to explore locomotion-related brain regions in which neuronal activities are affected by *l*-menthol administration. c-Fos, a translation product of the immediate early gene *c-fos*, is a marker of neuronal activation. Evaluation of c-Fos expression is useful for determining the brain regions that are activated by CNS drugs and those involved in specific behaviors [12, 13]. Accordingly, we employed c-Fos immunocytochemistry and conducted c-Fos expression mapping in the brain of mice to evaluate the ambulatory effects of *l*-menthol.

Locomotion involves neural activity involving numerous cortical and subcortical networks [14–16]. In particular, basal ganglia play key roles in controlling motor activities [17] and the locomotor effects of psychostimulants [18]. The striatum consists of medium-sized spiny neurons (≥95%) and interneurons [19, 20]. Striatonigral neurons almost exclusively express D1 dopamine receptors, and they project to the substantia nigra reticulata and internal globus pallidus/entopeduncular nucleus. Striatopallidal neurons, which almost exclusively express D2 dopamine receptors, project to the external globus pallidus [20, 21]. The rodent striatum is traditionally subdivided into two regions: dorsal (or neostriatum) and ventral [20]. Activities of both the striatonigral and striatopallidal neurons in the dorsal striatum correlate with locomotion, and neural clusters in the dorsal striatum are suggested to encode locomotion-relevant information [17, 22]. Also, D1 and D2 receptor-expressing neurons in the nucleus accumbens are involved in locomotion [23]. The dorsal striatum is densely innervated by dopamine neurons of the substantia nigra pars compacta, and the ventral striatum is densely innervated by dopamine neurons of the ventral tegmental area [20]. Excitatory glutamatergic neurons in the frontal cortex, including the primary motor cortex, orbital cortex, and medial prefrontal cortex (mPFC), project to the striatum, and they are involved in locomotion [24–27]. γ-Aminobutyric acid (GABA) projections from the motor cortex to the dorsal striatum are also involved in locomotion [28]. The dorsal raphe nucleus send neuronal projections to a variety of brain regions, and they can influence locomotion [29, 30]. The ventral pallidum in the basal forebrain that projects to the prefrontal cortex [31–33] can regulate locomotor speed [34]. The lateral hypothalamic area can influence activities in the mesolimbic dopamine pathway via projection to the ventral tegmental area [35–37]. Thus, neuronal activities in the lateral hypothalamic nucleus also influence locomotion [38]. The lateral septum nucleus (LS) is connected to the mesocorticolimbic dopamine system and the hippocampus [39], and the hippocampus-lateral septum pathway is involved in locomotion [40]. Neural projections from the median raphe nucleus (MnR) to the hippocampus and septum are also involved in locomotion [29, 41]. The locus coeruleus (LC) neurons project broadly to most of brain regions [42–44]. Thus, the activities of LC neurons can also influence locomotion [45]. As such, the present study examined c-Fos expression in these brain regions in mice administered *l*-menthol to investigate the brain regions in which neuronal activities were affected by *l*-menthol administration.

The previous study [8] showed that antagonists against dopamine receptors consistently attenuated the ambulatory effect of *dl*-menthol. Pretreatments with dopamine-depleting agents also attenuated the effect of *dl*-menthol. In addition, the dopamine transporter inhibitor bupropion, which promotes ambulation in mice, synergistically interacted with *dl*-menthol to induce mouse ambulation. These observations suggested that dopamine is involved in the ambulation-promoting effects of *dl*-menthol. However, the effects of menthol on dopamine in the brain are poorly known [46]. As such, the second aim of the present study was to examine whether *l*-menthol administration influences dopamine. To this end, *in vivo* microdialysis coupled with high-performance liquid chromatography (HPLC)/electrochemical detection was employed, and changes in the extracellular dopamine level in a brain region of a free-moving mouse was monitored after subcutaneous administration of *l*-menthol; the brain region examined in this experiment was chosen according to the results of the c-Fos mapping study.

## Materials and methods

### Subjects

Male ICR mice (Clea Japan, Tokyo, Japan) aged 7 to 15 weeks were used in the experiments. Mice were housed in aluminum cages (3 mice/cage) with a stainless-steel mesh top, and the cages contained wood shavings as bedding material for the mice. The mice had free access to commercial solid food (Clea Japan) and tap water. The cages were placed in a room artificially illuminated by fluorescent lamps on a 12-h light:12-h dark schedule (light period: 07:00–19:00), and the room temperature was set to 25°C ± 1°C. All experiments were conducted during the light period.

All animal experiments were approved (AE-16-10, AE-17-03, AE-18-02, AE-18-03) by the Ethics Committee for Experimental Animals of the National Institute for Environmental Studies, Japan, in accordance with the Guidelines for Proper Conduct of Animal Experiments (Science Council of Japan, 2006. http://www.scj.go.jp/ja/info/kohyo/pdf/kohyo-20-k16-2e.pdf).

### Chemical agents

*l*-Menthol was purchased from Nacalai Tesque (Kyoto, Japan). Before being subcutaneously administered, the *l*-menthol was mixed with a small amount of polyoxyethylene sorbitan monooleate (Tween 80; Nacalai Tesque), and diluted in a 0.9% NaCl (Nacalai Tesque) solution (saline).

Peppermint essential oil, of which a major constituent is menthol (35–55%), is used for medicinal purposes, and oral dosage of peppermint essential oil usually ranges from 187 to 500 mg/time, two or three times a day [47]. Also, *l*-menthol produces clinical effects after intragastrical administration at dose range of 80 to 320 mg [48]. Peak plasma level of menthol glucuronide, a metabolite of *l*-menthol, in human after intragastrical administration of 320 mg is comparable to that in rats after intragastrical administration of 400 mg/kg [49, 50]. *dl*-Menthol promotes mouse ambulation at 100 mg/kg when administered intraperitoneally and at 400 to 800 mg/kg when administered subcutaneously [3, 8]. Thus, the present study examined effects of subcutaneous administration of 100 to 800 mg/kg *l*-menthol.

### Evaluation of the ambulatory effect and preparation of brain samples for immunocytochemistry

The ambulatory activity of mice was measured using an ambulometer (SAM-10; O’Hara and Co., Tokyo, Japan) that was based on a tilting-cage method [51]. Each bucket-like activity cage (20 cm in diameter) of the apparatus is supported by a fulcrum in the center of the bottom. The fulcrum tilts according to movement of the mouse in the activity cage. The tilting movement of the cage activates three micro-switches that surround the cage. The number of activations of micro-switches during a set time is recorded as the ambulatory activity of the mouse.

Individual mice were placed in the activity cage, and 30 min later, saline was subcutaneously administered, followed by the measurement of ambulatory activity for 60 min. After the measurement, the mice were returned to their home cages. This procedure was repeated every day for 3 days to reduce stress to the mice (acclimation sessions). On the 4^th^ day (challenge session), individual mice were placed in an activity cage. After 30 min of adaptation, saline or 400 mg/kg *l*-menthol was subcutaneously administered in accordance with the previous study [8], and ambulatory activity was measured for 60 min. Immediately after the end of the ambulatory measurement, the mice were deeply anesthetized by intraperitoneal administration of 70–80 mg/kg pentobarbital (Nembutal^®^; Dainippon Sumitomo Pharma Co., Ltd., Osaka, Japan), and perfused transcardially with saline containing heparin (Wako Pure Chemical Industries, Ltd., Osaka, Japan), that euthanized the animals, followed by Speh’s fixative (4% paraformaldehyde, 0.2% saturated picric acid, and 0.05% glutaraldehyde in 0.1 M phosphate buffer (pH 7.4); Nacalai Tesque)). Brains were removed and post-fixed in the same fixative overnight at 4°C. Then, they were soaked in 0.1 M phosphate buffer (pH 7.4) containing 25% sucrose for cryoprotection until they had completely sunk. Brains were individually frozen using methyl butane cooled by dry ice and stored at −80°C.

### Immunocytochemistry for c-Fos

Coronal sections of brains from the olfactory bulb to the midbrain were cut at a thickness of 50 μm using a cryostat. Immunocytochemistry was performed on free-floating sections. After washing with Tris-buffered saline (TBS, pH 7.4; Nacalai Tesque), sections were incubated at 4°C with the primary antibody (F7799, Anti-c-Fos rabbit IgG; Sigma-Aldrich, Tokyo, Japan; 1:5000) in antibody diluent (TBS containing 0.25% λ-carrageenan, 1% bovine serum albumin, and 0.3% Triton X-100 (all from Sigma-Aldrich)) with 0.1% sodium azide (Nacalai Tesque) for 3 days. Sections were then washed in TBS and incubated at room temperature in antibody diluent containing biotinylated secondary goat anti-rabbit IgG (Vector Labs, Burlingame, CA, USA; 1:500) for 60 min. After washing with TBS, sections were incubated with ABC complex (ABC Elite kit; Vector Labs; 1:750) in antibody diluent for 90 min. Then, sections were reacted with H_2_O_2_ and diaminobenzidine (Sigma-Aldrich) to visualize the immunostaining. Sections were mounted onto subbed slides, allowed to air dry, dehydrated, and coverslipped using Permount (Sigma-Aldrich).

**Fig 1:**
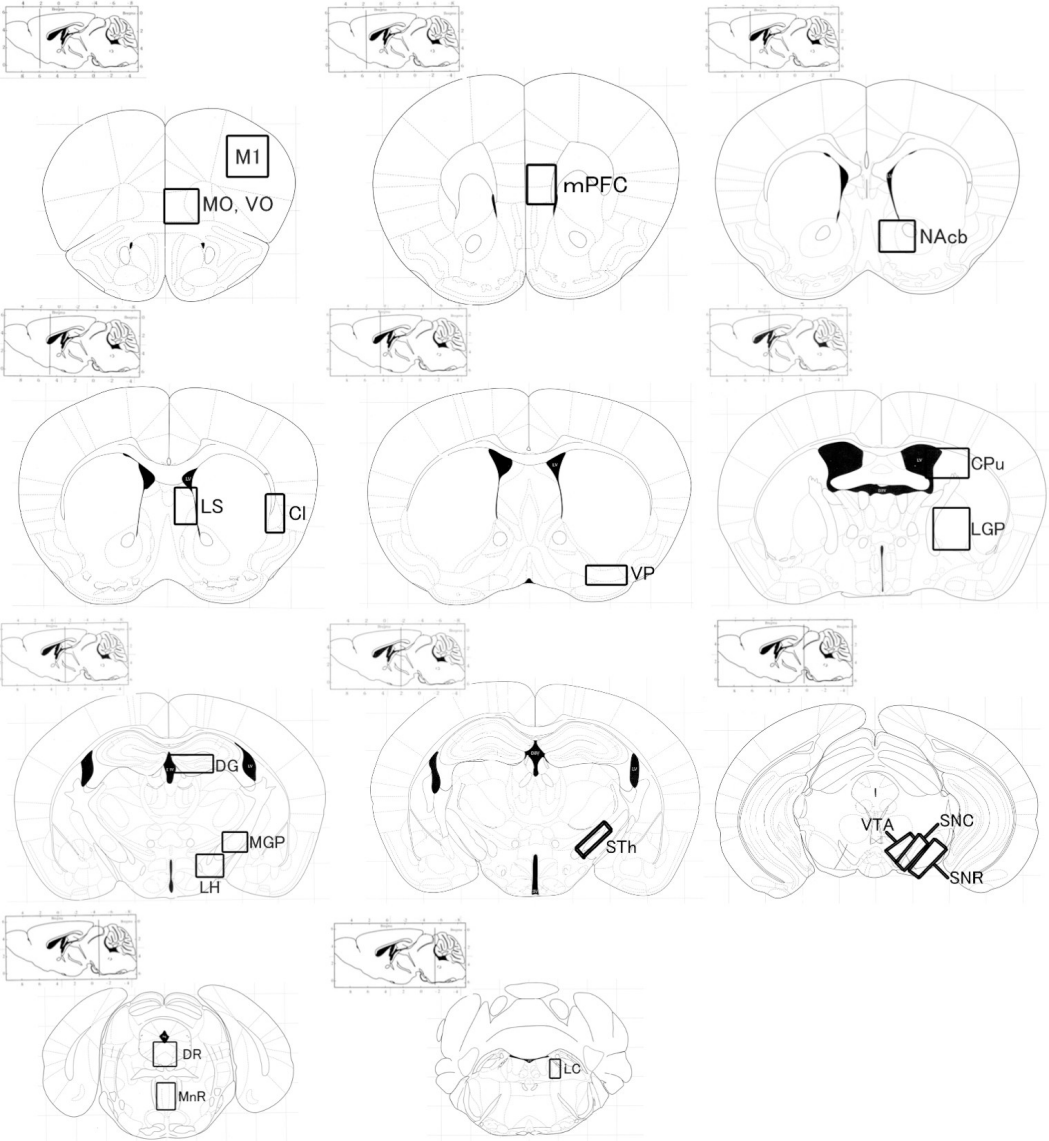
Brain regions examined in this study (Paxinos and Franklin, 2004). Abbreviations: MO: medial orbital cortex; VO: ventral orbital cortex; M1: primary motor cortex; mPFC: medial prefrontal cortex; LS: lateral septum nucleus; Cl: claustrum; NAcb: nucleus accumbens; VP: ventral pallidum; LGP: lateral globus pallidus; CPu, caudate putamen; DG: dentate gyrus; MGP: medial globus pallidus; LH: lateral hypothalamus; STh: subthalamic nucleus; VTA: ventral tegmental area; SNC: substantia nigra, pars compacta; SNR: substantia nigra, pars reticulate; DR: dorsal raphe nucleus; MnR: median raphe nucleus; LC: locus coeruleus.

The numbers of c-Fos-like immunoreactive (c-Fos-IR) nuclei in the 19 brain regions involved in locomotion (Fig 1) were quantified.

A camera (DFC490; Leica, Wetzlar, Germany) with an interface connected to a personal computer was used to obtain microscopic images of stained sections. Captured images at a magnification of 100× were printed in color and used for the quantification of c-Fos expression. Areas of 0.8 mm^2^ were manually counted for all brain regions except for the dentate gyrus (DG), in which the sizes of the areas for counting ranged from 0.11 to 0.27 mm^2^ due to its morphology.

### Microdialysis probe implantation

The brain microdialysis probe (D-I-6-02, cut-off: 50,000 Da; Eicom, Kyoto, Japan) was implanted in the mouse brain as previously reported [52, 53]. Briefly, mice were anesthetized by intraperitoneal administration of 50 mg/kg pentobarbital (Nembutal^®^). At the same time, 4 mg/kg carprofen (Rimadyl^®^; Zoetis Japan, Tokyo, Japan), an analgesic agent, was subcutaneously administered. The anesthetized mouse was fixed in a stereotaxic apparatus equipped with a mouse adapter (David Kopf, Tujunga, CA, USA). Based on the results of the c-Fos immunocytochemistry study, the brain dialysis probe was implanted into the dorsal striatum (AP: +0.1 mm, ML: +2.0 mm, DV: −2.8 mm) according to the mouse brain atlas [54] and fixed with dental cement.

Carprofen was used to control pain associated with the brain probe implantation. After the end of the probe implantation, the mice were gently returned to their home cage, and kept completely at rest. As pentobarbital is a short-acting type anesthetic, the animals recovered from anesthesia in a relatively short time. The condition of the animals was carefully observed until the microdialysis experiment.

### On-line measurement of dopamine

Online measurement of the extracellular dopamine level was performed as previously reported [51] 2 to 3 days after the implantation of the brain microdialysis probe. The probe-implanted mouse was placed in a cage for the microdialysis experiment and was allowed to move freely. Food and water were available *ad libitum* throughout the microdialysis measurement. Ringer’s solution (147 mM Na^+^, 4 mM K^+^, 2.3 mM Ca^2+^, and 155.6 mM Cl^−^) was perfused at a rate of 2 μL/min through the brain probe using a syringe pump (ESP-64; Eicom). Dialysate samples were collected every 25 min using an auto-injector (EAS-2; Eicom). The auto-injector automatically injected the dialysate sample into an HPLC system immediately after the end of each 25-min collection.

Dopamine in the dialysate samples was measured by HPLC (HTEC-500; Eicom) using an SC-50DS column (Eicom). The flow rate of the mobile phase (pH 3.5; 83% 0.1 M acetic acid-citric acid buffer, 17% methanol (Nacalai Tesque), 190 mg/L octanesulfonic acid (Nacalai Tesque), and 5 mg/L Na_2_EDTA (Wako Pure Chemical Industries Ltd.)) was 0.23 mL/min. An electrochemical detector (ECD-300; Eicom) with a graphite electrode (WE-3G; Eicom) was used to detect dopamine. The applied voltage was +700 mV against a Ag/AgCl reference electrode. Data were collected from the HPLC system via an interface (EPC-300; Eicom) connected to a personal computer. PowerChrom software (AD Instruments Japan, Nagoya, Japan) was used to analyze chromatograms.

Immediately after the end of the dopamine measurement, the mouse was euthanized by intraperitoneal administration of lethal dose of pentobarbital (Nembutal^®^).

### Statistical analyses

#### Ambulatory activity

To eliminate differences in baseline ambulatory activity, the ambulatory activity of each mouse at each time point was divided by the total activity of the mouse during the 30-min adaptation period before the administration of drugs, followed by normalization using square root transformation. The data were analyzed using two-way analysis of variance (ANOVA), followed by Tukey’s test.

#### c-Fos-IR

Differences in the number of c-Fos-IR nuclei in each brain region between saline-administered and *l*-menthol-administered mice were analyzed using the Wilcoxon test. If a significant difference was observed, the relationship between c-Fos-IR in the brain region and ambulatory activity was examined using a single regression analysis. For the regression analysis, the c-Fos-IR data and the ambulatory activity were normalized using square root transformation. The least-squares method was used to determine the best-fit regression equation, and the significance of the determined single regression equation was evaluated by ANOVA.

#### Dopamine

For each mouse, the measured value of dopamine in the dialysate sample just before the administration of saline or *l*-menthol was defined as the baseline value, and the measured values before and after the administration are expressed as ratios to the baseline value.

Changes in the dopamine level in the dialysate samples after the administration of saline or *l*-menthol were analyzed using repeated measures ANOVA. The statistical significance at each time point was examined by one-way ANOVA followed by Tukey’s test. The dose-response relationship based on the area under the curve (AUC) was analyzed by one-way ANOVA followed by Tukey’s test.

## Results

### Effects of the subcutaneous administration of *l*-menthol on ambulatory activity and c-Fos expression in the brain

**Fig 2:**
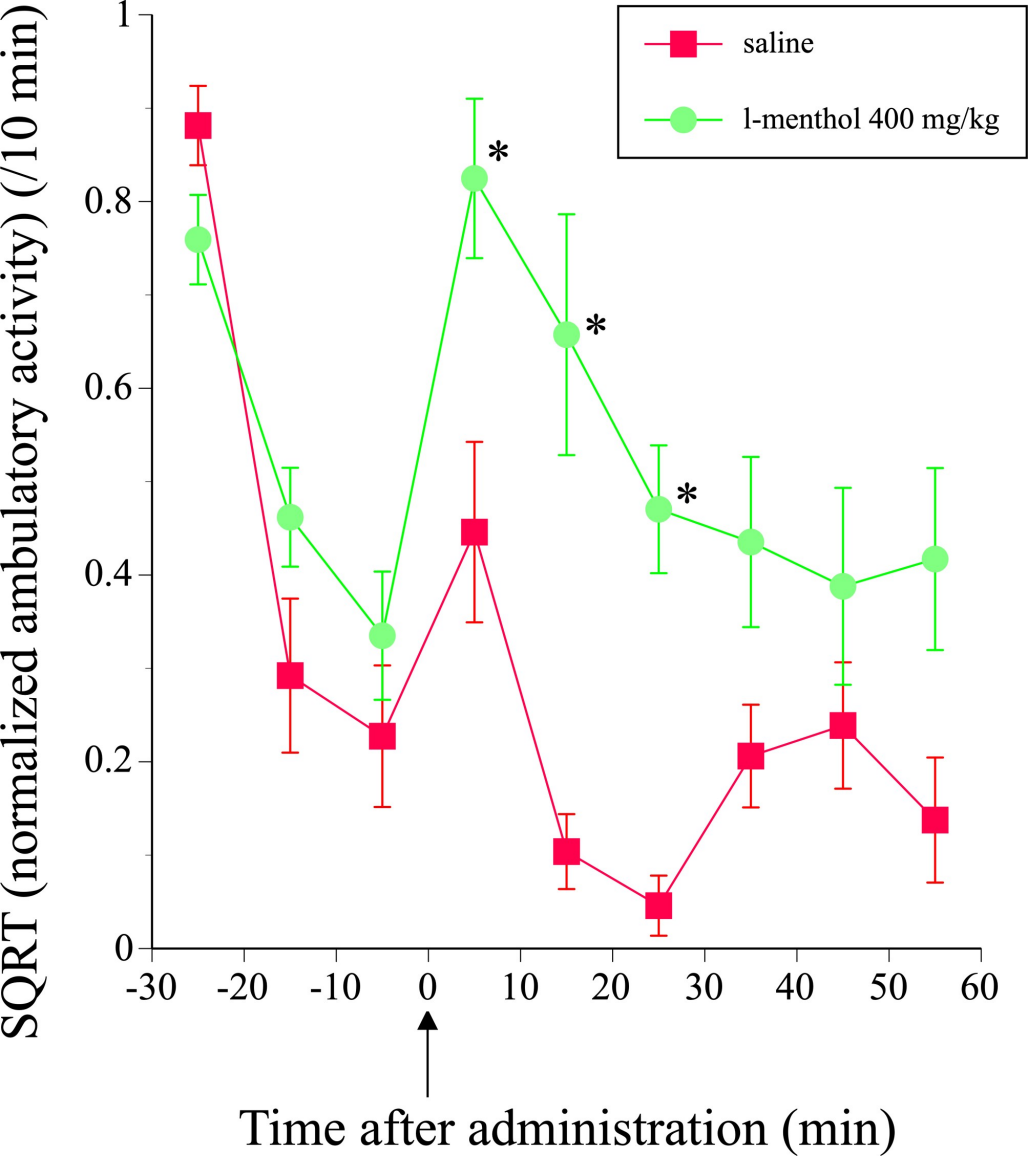
Time course of ambulatory activity before and after the subcutaneous administration of saline or 400 mg/kg *l*-menthol on the 4^th^ day (challenge session). All mice were administered saline and the ambulatory activity was measured on the 1^st^ to 3^rd^ days (acclimation sessions). On the 4^th^ day, 30 min after introduction into the activity cages, one group was administered saline (N = 7), the other group was administered 400 mg/kg *l*-menthol (N = 11), and the ambulatory activity was then measured for 60 min. The arrow indicates the time point of the administration. Immediately after the end of the ambulatory measurements, the mice were transcardially perfused with fixative, and their brains were collected for later use in c-Fos immunocytochemistry. Symbols show the mean values of the square-root- transformed ambulatory activity, that was normalized using the total ambulatory activity during the 30 min adaptation period before the administration, for each 10-min period that were plotted against the midpoint of the measurement period, and vertical lines denote the standard errors of the means. *P < 0.05 compared with the vehicle control by Tukey’s test. The data are presented in S1 Data).

Although the acclimation procedure was conducted for 3 consecutive days to reduce stress in the mice, the experimental environment and handling still stimulated ambulatory activity on the 4^th^ day (challenge session). When the mice were introduced to the activity cages on the 4^th^ day, the animals showed high ambulatory activity that gradually decreased (Fig 2). After 30 min of adaptation, saline or 400 mg/kg *l*-menthol was subcutaneously administered to the mice. The ambulatory activity slightly and temporarily increased immediately after the saline administration, probably due to the stimulation from the injection. However, *l*-menthol administration had a much larger effect on the ambulatory activity: it significantly increased the ambulatory activity immediately after administration, and the effect persisted for almost 60 min (2-way ANOVA: dose (F(1, 16) = 12.8583, P = 0.0025; time (F(5, 12) = 3.6462918, P = 0.0011; Fig 2), consistent with the results of a previous study [8].

**Fig 3:**
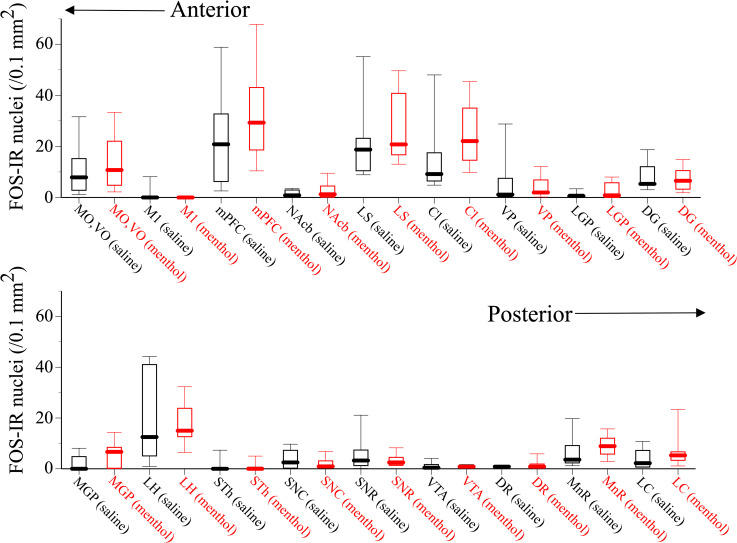
Numbers of c-Fos-IR nuclei in 18 brain regions of mice that were administered saline or 400 mg/kg *l*-menthol. Data are shown as box plots (saline; N = 7, menthol; N = 11). The data are presented in S2 Data. Abbreviations for the 18 brain regions in the figure: MO and VO: medial orbital cortex and ventral orbital cortex, respectively; M1: primary motor cortex; mPFC: medial prefrontal cortex; LS: lateral septum nucleus; Cl: claustrum; NAcb: nucleus accumbens (including both the core and shell); VP: ventral pallidum; LGP: lateral globus pallidus; DG: dentate gyrus; MGP: medial globus pallidus; LH: lateral hypothalamus; STh: subthalamic nucleus; VTA: ventral tegmental area; SNC: substantia nigra, pars compacta; SNR: substantia nigra, pars reticulate; DR: dorsal raphe nucleus; MnR: median raphe nucleus; LC: locus coeruleus.

The expression of c-Fos in the brains of these mice was examined. c-Fos-IR nuclei in the 19 brain regions (Fig 1) were quantified, and the numbers were compared between saline-administered control mice and *l*-menthol-administered mice. Fig 3 shows the numbers of c-Fos-IR nuclei in the 18 brain regions other than the dorsal striatum (caudate putamen (CPu)) of the mice administered saline or 400 mg/kg *l*-menthol. c-Fos-IR nuclei were observed in these brain regions. The numbers of c-Fos-IR nuclei differed among the 18 brain regions: relatively large numbers of c-Fos-IR nuclei were observed in the orbital cortex (medial (MO) and ventral (VO)), mPFC, LS, claustrum (Cl), DG, lateral hypothalamus (LH), MnR, and LC in both the saline-administered and *l*-menthol-administered mice. However, no significant differences were observed in the number of c-Fos-IR nuclei in the 18 brain regions between the mice administered saline and the mice administered *l*-menthol.

**Fig 4:**
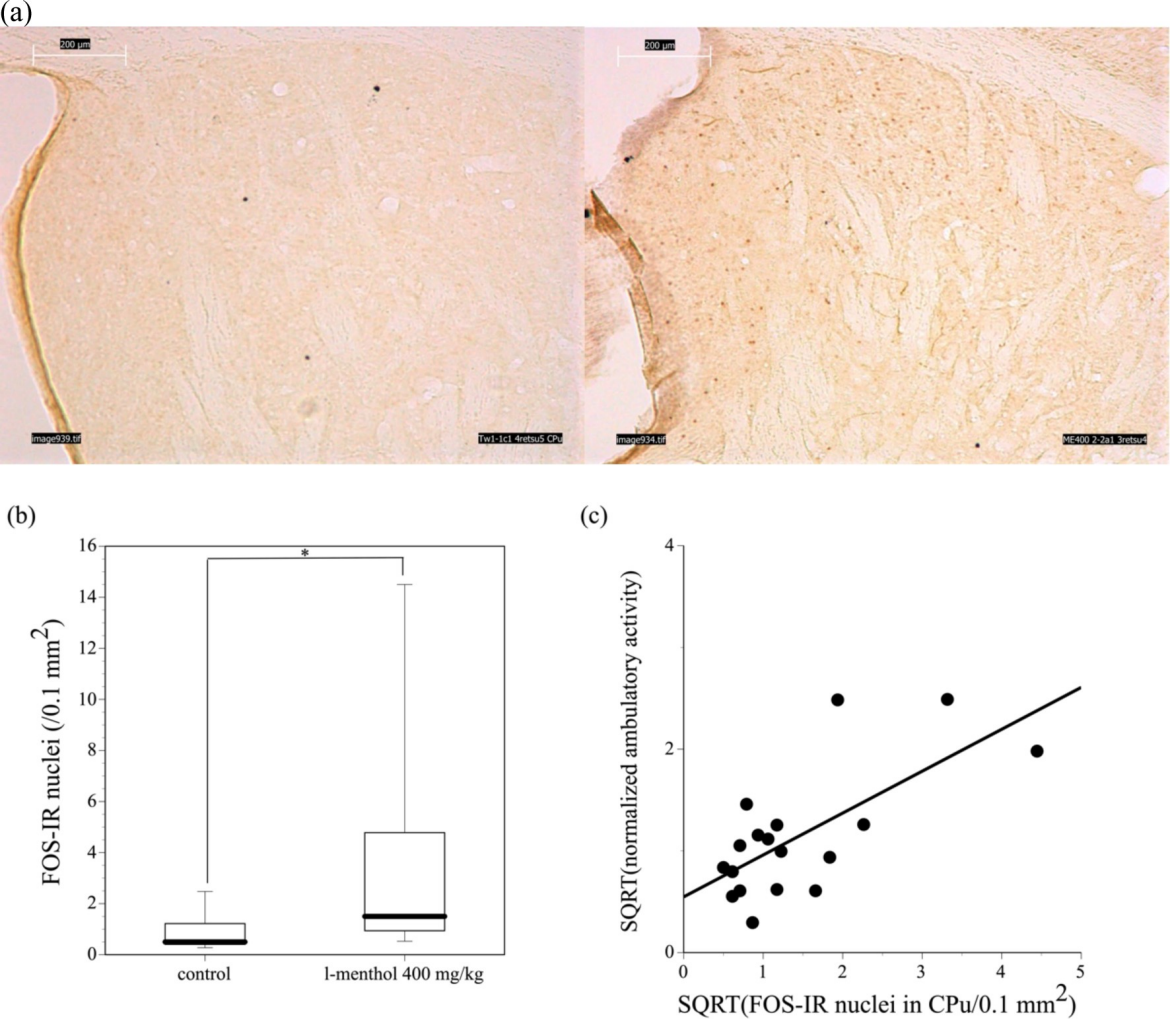
c-Fos-IR nuclei in the dorsal striatum (caudate putamen (CPu)) of mice administered saline or 400 mg/kg *l*-menthol. (a) Immunostaining for c-Fos in the dorsal striatum of a mouse administered saline (left panel) and a mouse administered 400 mg/kg *l*-menthol (right panel). Dark brown dots indicate c-Fos-IR-positive nuclei. Scale bars: 200 μm. (b) Number of c-Fos-IR nuclei in the dorsal striatum of mice administered saline or *l*-menthol. Data presented in S2 Data are shown as box plots.*P < 0.05. N = 7 to 11. (c) Relationship between ambulatory activity and c-Fos-IR nuclei in the dorsal striatum. The best-fit regression lines were determined using the least-squares method.

In contrast, the number of c-Fos-IR nuclei in the dorsal striatum, i.e., the CPu, of the mice administered *l*-menthol was much higher than that of the mice administered saline (Fig 4A). The number of c-Fos-IR nuclei in the dorsal striatum of mice administered *l*-menthol was significantly higher than that of mice administered saline (χ^2^_1_ = 4.5438, P = 0.033; Fig 4B).

The correlation between the number of c-Fos-IR nuclei in the dorsal striatum and the ambulatory activity was examined using a single regression analysis (Fig 4C). The least-squares method was used to determine the best-fit regression line shown in the figure, and the equation was as follows:

SQRT(normalized ambulatory activity) = 0.548 + 0.41 × SQRT(c-Fos-IR in the dorsal striatum), where SQRT indicates square root transformation.

The equation indicated statistical significant (F(1, 16) = 14.1107; P = 0.0017). The coefficient of determination (R^2^) was calculated to be 0.469.

Collectively, subcutaneous administration of *l*-menthol increased the ambulatory activity and the number of c-Fos-IR nuclei in the dorsal striatum of mice. The degree of the ambulatory activity was positively and significantly correlated with the number of c-Fos-IR nuclei in the dorsal striatum.

### Effect of the subcutaneous administration of *l*-menthol on extracellular dopamine in the dorsal striatum

**Fig 5:**
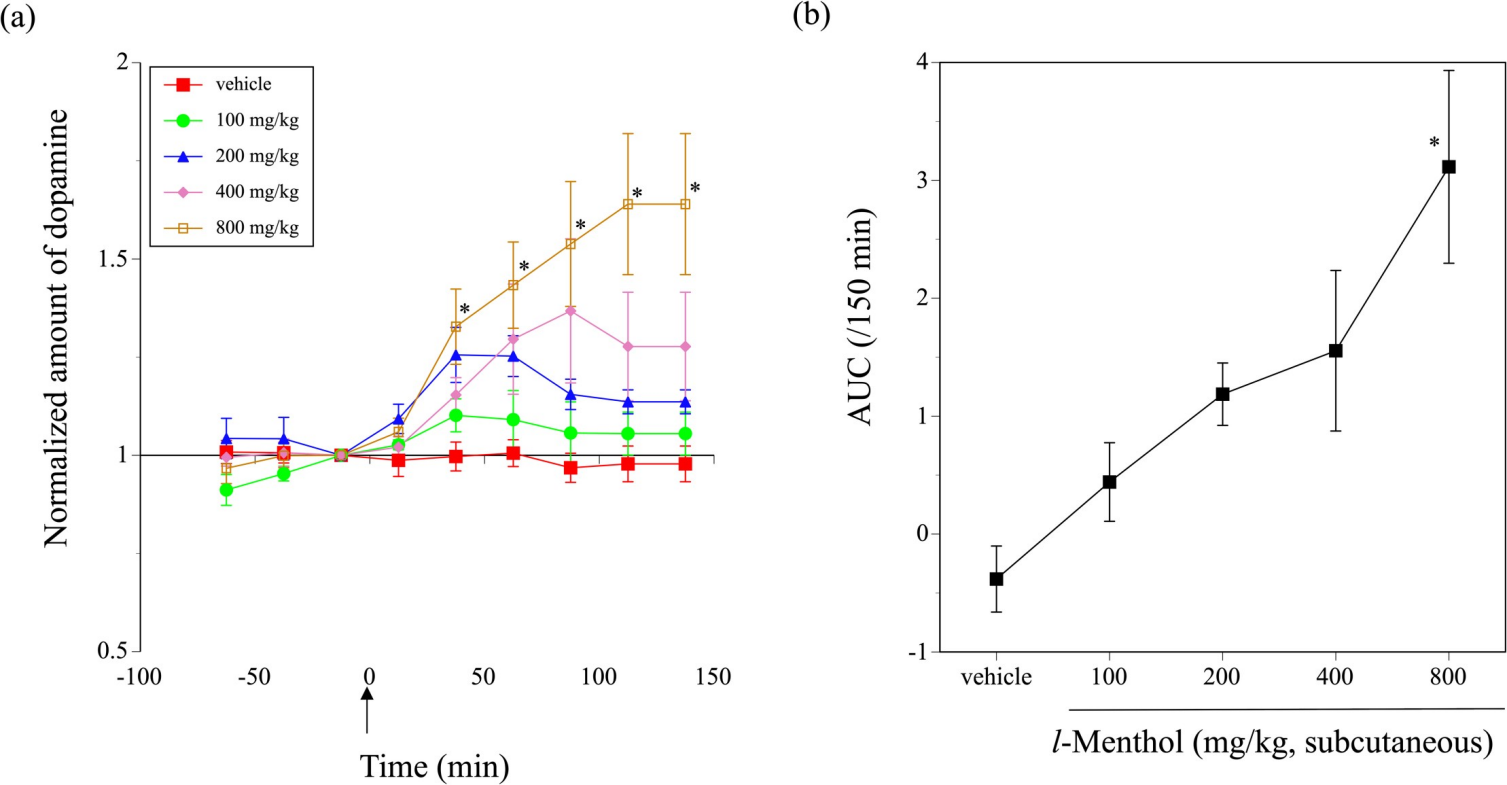
Effects of the subcutaneous administration of 100 to 800 mg/kg *l*-menthol on the extracellular dopamine level in the dorsal striatum of free-moving mice. (a) Changes in the dopamine level in the dialysates obtained from the dorsal striatum of mice administered saline or 100 to 800 mg/kg *l*-menthol. The data are presented in S3 Data. (b) The dose-response relationship for the effect of *l*-menthol on the dopamine level as evaluated using the AUC. *P < 0.05 compared with saline by Tukey’s test. N = 10 to 16.

Fig 5A shows the changes in the normalized dopamine levels in the dialysate samples from the dorsal striatum before and after the subcutaneous administration of saline or 100 to 800 mg/kg *l*-menthol. While the dopamine level remained almost constant after saline administration, the levels significantly increased after the administration of the *l*-menthol doses (repeated-measures ANOVA: dose F(4, 58) = 4.3489, P = 0.0038, and time F(6, 53) = 6.8113, P < 0.0001). The effects became significant at 37.5 min, and the significance remained until 137.5 min after *l*-menthol administration (one-way ANOVA: 37.5 min, F(4, 58) = 3.6216, P = 0.0106; 62.5 min, F(4, 58) = 2.8455, P = 0.0319; 87.5 min, F(4, 58) = 3.423, P = 0.014; 112.5 min, F(4, 58) = 5.3985, P = 0.0009; and 137.5 min, F(4, 58) = 4.1384, P = 0.0051). Analysis of the AUC showed that the effects of *l*-menthol were dose-dependent (F(4, 58) = 5.2151, P = 0.0012; Fig 5B).

Collectively, the subcutaneous administration of 100 to 800 mg/kg *l*-menthol dose-dependently enhanced the extracellular dopamine level in the dorsal striatum of mice.

## Discussion

The current study provides novel information regarding the CNS-activating effects of *l*-menthol. First, subcutaneous administration of *l*-menthol significantly increased the number of c-Fos-IR-positive nuclei in the dorsal striatum of mice, which promoted ambulatory activity in the mice; a positive correlation was seen between the number of c-Fos-IR-positive nuclei and the degree of ambulatory activity. Second, subcutaneous administration of *l*-menthol dose-dependently increased the extracellular dopamine level in the dorsal striatum of mice. Taken together, these observations show that after subcutaneous administration, *l*-menthol increases extracellular dopamine and c-Fos expression levels in the dorsal striatum, and promotes ambulation in mice.

c-Fos-IR nuclei were observed in the examined brain regions, and relatively larger numbers of c-Fos-IR nuclei were observed in the orbital cortex, mPFC, LS, Cl, DG, LH, MnR, and LC without significant differences between the mice administered saline and those administered *l*-menthol. Given that previous studies have reported that *c-fos* and/or c-Fos expression is observed in the cerebral cortex, LS, Cl, hippocampus, MnR, and LC of rodents that are placed in an open field or a novel chamber [55–60], the results of the current study suggest that both the experimental environment and manipulations induce c-Fos in these brain regions. The experimental environment and manipulations also stimulated ambulatory activity in mice. Given that these brain regions are involved in locomotion, it is likely that the activation of these brain regions was involved in promoting ambulatory activity in the mice after they were moved from their home cages to the activity cage. However, whether these brain regions were involved in the ambulation-promoting effect of *l*-menthol remains unclear, as no significant difference was observed in the number of c-Fos-IR nuclei in these brain regions between the mice administered saline and those administered *l*-menthol. Similarly, in the present study, there was no significant difference between the mice administered saline and those administered *l*-menthol in the number of c-Fos-IR nuclei in other brain regions, such as the nucleus accumbens, ventral pallidum, lateral globus pallidus, medial globus pallidus, subthalamic nucleus, ventral tegmental area, substantia nigra pars compacta, substantia nigra pars reticulate, and dorsal raphe nucleus, even though these brain regions are also involved in locomotion. Thus, elucidation of the roles of these brain regions in the ambulatory effect of *l*-menthol must await future research.

Previous studies have shown that menthol has inhibitory effects on neuronal activity [9, 10, 61–64], however, to the best of our knowledge, activating effects of menthol on neuronal activity are poorly known. The present study revealed that the administration of *l*-menthol increased the number of c-Fos-IR-positive nuclei in the dorsal striatum of mice. This result suggests that *l*-menthol administration activates neural activity in the dorsal striatum. In addition, the mice administered *l*-menthol exhibited significantly enhanced ambulatory activity, and the degree of ambulatory activity was positively correlated with the number of c-Fos-IR-positive nuclei in the dorsal striatum. As the activities of neurons in the dorsal striatum are correlated with locomotion [17, 22], our results suggest that the activation of neuronal activity in the dorsal striatum is involved in the ambulation-promoting effect of *l*-menthol.

Neurotropic factors, neurotransmitters, depolarization, increased Ca^2+^ influx, and elevated levels of intracellular/intranuclear Ca^2+^ are major factors for the induction of *c-fos* [55]. Menthol can influence Ca^2+^ channels, and previous studies have consistently reported that menthol inhibits Ca^2+^ channels [7]. N-methyl-D-aspartate (NMDA) glutamate receptors that have a Ca^2+^ channel are expressed in striatal neurons [65]. Although it remains unclear how *l*-menthol influences the activity of NMDA receptors, a binding assay study [66] suggested that the potency of *l*-menthol for affecting NMDA receptors is very low. The striatum includes GABA-containing interneurons [20], and it also receives GABAergic input from the cerebral cortex [28]. Although *l*-menthol can influence the function of GABA_A_ receptors [7], it likely inhibits neuronal activity rather than promotes the activity by enhancing the GABA_A_ receptor-mediated inhibitory currents [64, 67]. The striatum also contains acetylcholine-containing interneurons, and it receives cholinergic input from other brain regions [68, 69]. The previous studies [70–72] show that menthol acts on nicotinic cholinergic receptors as an negative allosteric modulator, suggesting that menthol may not activate neuronal activity through nicotinic cholinergic receptors. Although it remains unclear how *l*-menthol influences cholinergic neurotransmission through muscarinic cholinergic receptors, the binding assay study [66] suggested that the potency of *l*-menthol for affecting muscarinic cholinergic receptors (M1 to M5) is also very low. However, further studies are needed to fully understand the roles of these receptors and channels in the induction of c-Fos by *l*-menthol in striatal neurons.

Medium-sized spiny neurons that express dopamine D1 or D2 receptors constitute the vast majority (≥95%) of striatal neurons; thus, dopamine is a principal neurotransmitter in the striatum. The binding assay study [66] suggested that the action of *l*-menthol on these dopamine receptors is should be presumed to be very weak. However, the present study demonstrated that the administration of *l*-menthol increased the extracellular dopamine level in the dorsal striatum. As dopamine can induce *c-fos* through dopamine receptors [73–75], the enhancement of dopamine-mediated neurotransmission is likely one of the causes of the effect of *l*-menthol on c-Fos expression in the striatum. The binding assay study also showed that *l*-menthol inhibits the binding of [^3^H]-WIN35,428 to the dopamine uptake/binding site of dopamine transporter; this is similar to the action of the dopamine transporter inhibitor GBR12909, and suggests that *l*-menthol inhibits the binding of dopamine to the dopamine transporter, thereby leading to decreased dopamine uptake [66]. The action of *l*-menthol on the dopamine transporter would also account for the synergistic interaction with the ambulation-promoting effect of the dopamine transporter inhibitor bupropion, which also enhances the extracellular dopamine level and increases the number of c-Fos-IR nuclei in the striatum of mice [51]. In addition, *l*-menthol may also act on adenosine A2a receptors [66]. Adenosine A2a receptors interact with dopamine receptors. The locomotion-stimulating effect of caffeine is produced via antagonism of adenosine A2a receptors expressed in the medium-sized spiny neurons in the striatum through a dopamine-dependent mechanism [76]. Accordingly, the adenosine A2a receptors may also be involved in the effects of *l*-menthol on c-Fos expression in the striatum and ambulation.

Not only the nigrostriatal system, but also the mesolimbic and mesocortical systems are major dopaminergic systems in the brain. In addition, adenosine A2a receptors are highly expressed in the dopamine-rich regions of the brain. *l*-Menthol may potentially influence the mesolimbic and mesocortical dopaminergic systems through dopamine transporters and adenosine A2a receptors. As *l*-menthol did not influence c-Fos expression in the brain regions related to the mesolimbic and mesocortical dopaminergic systems, we did not examine the effects of *l*-menthol on dopamine in those brain regions in the current study. It is noteworthy that *l*-menthol can act on not only dopamine transporters and adenosine A2a receptors, but also on other kinds of neurotransmitter receptors and channels at the same pharmacologically relevant concentration range [7, 66]. Thus, different and complex mechanisms are likely responsible for the effects of *l*-menthol on c-Fos expression in different brain regions. Whether *l*-menthol influences dopamine-mediated neurotransmission in the brain regions related to the mesolimbic and mesocortical dopaminergic systems remains to be examined in future studies.

## Conclusion

The present study revealed novel information regarding the CNS-activating effects of *l*-menthol. Namely, the results showed that after subcutaneous administration, the *l*-menthol enhances dopamine-mediated neurotransmission, and activates neuronal activity in the dorsal striatum, thereby promoting ambulation in mice.

## Supporting information

S1 Data (DOCX)

S2 Data (DOCX)

S3 Data (DOCX)
