# Supplementary material for: l-Menthol increases extracellular dopamine and c-Fos-like immunoreactivity in the dorsal striatum, and promotes ambulatory activity in mice
Source: PLoS One. 2021 Nov 30;16(11):e0260713. doi: 10.1371/journal.pone.0260713 (PMC8631625; doi:10.1371/journal.pone.0260713)
Supplement: Supplementary file 1 [file pone.0260713.s001.docx]

saline control

time (min)	-25	-15	-5	5	15	25	35	45	55
animal No.									
1	0.859726953621095	0.466252404	0.208514414	0	0	0	0.208514414	0.208514414	0
2	0.723746864455746	0.422577127	0.545544726	0.46291005	0.243975018	0.21821789	0.21821789	0	0
3	1	0	0	0.707106781	0	0	0	0.316227766	0.316227766
4	0.950923403407938	0.230632802	0.206284249	0.778705964	0.206284249	0.103142125	0.272888411	0.461265604	0.399467731
5	1	0	0	0.40824829	0	0	0.466849781	0	0
6	0.88578517972214	0.411376676	0.214834462	0.429668924	0.175411604	0	0.175411604	0.350823208	0
7	0.749149177264394	0.515078754	0.416496564	0.335029697	0.101015254	0	0.101015254	0.335029697	0.24743583
mean	0.881333082638759	0.292273966	0.227382059	0.445952815	0.103812304	0.045908574	0.206128194	0.238837241	0.13759019
SE	0.0424822467899706	0.082479812	0.07580607	0.09661882	0.04012224	0.032184056	0.055003376	0.06765871	0.066954415

menthol

time (min)	-25	-15	-5	5	15	25	35	45	55
animal No.									
1	0.671550736844851	0.242535625	0.700140042	1	0.280056017	0.198029509	0.140028008	0.420084025	0.140028008
2	0.87461835388772	0.480384461	0.065372045	0.960768923	0.92911133	0.363976143	0.160128154	0	0.428673167
3	0.867897893330058	0.377964473	0.322329186	0.82964552	0.875347797	0.469871494	1.145289619	1.243163121	1.289316742
4	0.856841417760529	0.22501758	0.463885625	0.43574467	0.22501758	0.251577303	0.515580047	0	0.251577303
5	0.729703729240527	0.496742636	0.469871494	0.852802865	0.483493778	0.592156525	0.360374985	0.322329186	0.161164593
6	0.739118594202782	0.673575314	0	0.739118594	1.065016084	0.827759135	0.769800359	0.735980072	0.649073414
7	0.855398922768302	0.51796977	0	1.489802737	1.634237347	0.897149959	0.455320846	0.382546028	0.349215148
8	0.842927230423525	0.350438322	0.40824829	0.614160388	0.486664263	0.546118681	0.296174439	0.310630372	0.386164223
9	0.892142571199771	0.349927106	0.285714286	0.534522484	0.285714286	0.349927106	0.515078754	0.24743583	0.24743583
10	0.350823207722812	0.794209815	0.496138938	0.91146543	0.464095481	0.372104204	0.214834462	0.411376676	0.429668924
11	0.669774686623137	0.57192902	0.473602223	0.703794787	0.502331015	0.30570892	0.216168851	0.193347298	0.255774433
mean	0.759163394909456	0.461881284	0.335027466	0.824711491	0.657371362	0.470398089	0.435343502	0.387899328	0.417099253
SE	0.0478950411712241	0.052939327	0.068649941	0.085371241	0.128981703	0.068428686	0.091138475	0.105444266	0.097463861
