# Supplementary material for: l-Menthol increases extracellular dopamine and c-Fos-like immunoreactivity in the dorsal striatum, and promotes ambulatory activity in mice
Source: PLoS One. 2021 Nov 30;16(11):e0260713. doi: 10.1371/journal.pone.0260713 (PMC8631625; doi:10.1371/journal.pone.0260713)
Supplement: Supplementary file 2 [file pone.0260713.s002.docx]

saline control FOS like immunorieactivity quantification (/0.1 mm2)

brain regio	MO,VO	M1	mPFC	NAcb	LS	Cl	VP	LGP	DG	MGP	LH	STh	SNC	SNR	VTA	DR	MnR	LC	CPu	SQRT (normalized ambulati
animal No.																				
1	12.875	0.13	20.83	3.54	23.91	20	0.278	0.833	11.197	0	12.5	0	8.4375	24.25	4.583	1.25	22.03	2.22	0.5	0.607492896293956
2	4.125	0	6.67	0.21	20.94	8.333	1.111	0.278	12.34	0	4.444	0	0.9375	2.75	0	0.42	4.69	7.22	0.75	0.294883912309794
3	16	0	28.33	0.21	9.38	10	9.444	3.889	5.0556	5.417	34.167	0	2.5	3.25	2.083	1.25	2.66	1.11	1.375	0.620173672946042
4	35.5	10.13	65	3.54	62.97	55	33.611	1.389	20.296	2.917	44.444	9.167	3.75	4	0.417	1.25	3.59	7.22	2.75	0.6076436202502
5	7.875	0	34.17	1.04	18.75	9.167	1.111	0.556	5.316	8.75	43.333	0	10	8.5	0	0.42	10.63	11.67	0.5	1.05184741228156
6	0.75	0	1.67	0	8.75	4.583	0.278	0	2.584	0	0	0	0	0	0	0.83	0.94	0.56	0.25	0.836660026534076
7	2.375	0	6.11	0.83	13.75	5.83	1.667	0	4.6939	0	6.944	0	0	0.75	0.417	0	2.19	0.56	0.375	0.553283335172488
median	7.875	0	20.83	0.83	18.75	9.167	1.111	0.556	5.316	0	12.5	0	2.5	3.25	0.417	0.83	3.59	2.22	0.5	
1st quartile	3.25	0	6.39	0.21	11.565	7.0815	0.6945	0.139	4.8748	0	5.694	0	0.4688	1.75	0	0.42	2.425	0.835	0.438	
3rd quartile	14.4375	0.065	31.25	2.29	22.425	15	5.5555	1.111	11.769	4.167	38.75	0	6.0938	6.25	1.25	1.25	7.66	7.22	1.063	

l-menthol FOS like immunorieactivity quantification (/0.1 mm2)

brain regio	MO,VO	M1	mPFC	NAcb	LS	Cl	VP	LGP	DG	MGP	LH	STh	SNC	SNR	VTA	DR	MnR	LC	CPu	SQRT (normalized ambulati
animal No.																				
1	10.75	0	40.83	4.17	24.38	26.25	1.616	1.389	11.33	1.25	14.722	0	0.9375	1.25	0	2.08	8.75	1.67	11	2.48893655901576
2	20.25	0	43.06	4.58	46.56	43.333	10.278	8.333	8.14	8.75	23.889	0.833	2.5	3.75	1.25	1.67	10.31	5	5.125	1.25881507165087
3	5.625	0	25	0.83	54.22	22.083	2.5	1.389	6.541	0.833	13.889	0	0.9375	1.5	1.25	0.83	9.06	8.89	3.375	0.936776932043143
4	8.735	0	24.72	0.42	20.78	17.917	0.278	0	15.781	7.5	23.889	3.333	0.625	2.75	0.833	0.83	5.94	5.56	0.625	1.45883690939225
5	46.875	0	83.61	5.83	41.25	48.333	14.722	7.778	14.311	19.58	45	7.5	8.125	11.5	2.083	7.5	13.75	6.11	19.75	1.98022632451351
6	24.25			3.13	39.38	22.917	2.5	0	6.5922	0	12.222	0							0.875	1.15470053837925
7	1.875	0	12.22	0	16.72	14.167	1.389	0	0.53	0	6.389	0	0.625	1.75	0.417	0	2.81	6.67	1.125	1.11607080200484
8	2.375	0	8.61	0.21	16.41	3.333	0	0.833	3.101	0	6.389	0	5	4.5	1.25	0.42	5.78	0.56	0.375	0.79555728417573
9	4.5	0	18.61	1.25	16.72	14.583	1.944	0	3.672	7.5	15	0	0.9375	1.25	0.833	0.42	2.81	5	1.375	1.25356634105602
10	11.125	0	33.61	0	19.38	14.583	1.944	0.833	2.7094	10.83	17.778	0	1.875	5	0		12.03	3.33	1.5	0.995316133501048
11	22.625	0	51.94	15	7.97	37.917	8.333	7.222	4.181	6.667	23.611	0		2.25	0.417		17.66	37.78	3.75	2.48409575244536
median	10.75	0	29.305	1.25	20.78	22.083	1.944	0.833	6.541	6.667	15	0	0.9375	2.5	0.833	0.83	8.905	5.28	1.5	
1st quartile	5.0625	0	20.138	0.315	16.72	14.583	1.5025	0	3.3865	0.417	13.055	0	0.9375	1.563	0.417	0.42	5.82	3.748	1	
3rd quartile	21.4375	0	42.503	4.375	40.315	32.084	5.4165	4.306	9.735	8.125	23.75	0.417	2.5	4.313	1.25	1.773	11.6	6.53	4.438	
