# Supplementary material for: l-Menthol increases extracellular dopamine and c-Fos-like immunoreactivity in the dorsal striatum, and promotes ambulatory activity in mice
Source: PLoS One. 2021 Nov 30;16(11):e0260713. doi: 10.1371/journal.pone.0260713 (PMC8631625; doi:10.1371/journal.pone.0260713)
Supplement: Supplementary file 3 [file pone.0260713.s003.docx]

Alteration of dopamine in the dorsal striatum

saline

time (min)	-62.5	-37.5	-12.5	12.5
animal No.				
1	0.961226994	0.879509202	1	0.631738241
2	1.04536862	0.981226778	1	0.974121635
3	0.990205835	0.964384853	1	1.093699262
4	0.993973973	1.00269248	1	1.05878582
5	0.97795813	1.085769621	1	1.043345937
6	1.158288043	1.171875	1	1.016304348
7	1.147373541	1.055308505	1	0.980544747
8	0.971100763	0.998703855	1	1.038452307
9	0.99824128	1.003810561	1	1.033290063
10	0.838704471	0.922580298	1	1.001721633
mean	1.008244165	1.006586115	1	0.987200399
se	0.029274306	0.026178632	0	0.041095609

l-menthol 100 mg/kg,sc

time (min)	-62.5	-37.5	-12.5	12.5
animal No.				
1	0.816172053	0.820016821	1	1.024029797
2	0.873320002	0.99158257	1	1.023066549
3	0.629146554	0.886926158	1	0.953630482
4	1.085559175	1.015128484	1	1.044010134
5	0.790207128	0.918595797	1	1.040207128
6	0.9135556	0.976043122	1	1.060491116
7	1.002911956	0.993362453	1	1.012161699
8	1.044460466	1.022750941	1	0.946687495
9	0.951036581	0.981815609	1	1.067218566
10	0.991363159	0.910844034	1	1.067129735
11	0.932462897	0.970975014	1	1.055513808
mean	0.911835961	0.953458273	1	1.026740592
se	0.039277044	0.018717045	0	0.01268618

l-menthol 200 mg/kg,sc

time (min)	-62.5	-37.5	-12.5	12.5
animal No.				
1	1.181638697	1.039075354	1	1.044998355
2	0.968970737	0.991058415	1	1.034196659
3	0.941615843	0.996436648	1	1.15199068
4	1.245227867	1.036228354	1	1.050135081
5	1.323704333	1.748709235	1	1.459251029
6	0.890235388	0.917314981	1	0.98673449
7	0.685779203	0.699812884	1	1.425287356
8	0.809829559	0.918398319	1	1.025332711
9	0.906120571	0.957892315	1	1.046019328
10	1.031772466	1.098354232	1	0.986024033
11	1.19958635	1.089451913	1	0.999482937
